# Measurement System of a Magnetic Suspension System for a Jet Engine Rotor †

**DOI:** 10.3390/s20030862

**Published:** 2020-02-06

**Authors:** Paulina Kurnyta-Mazurek, Artur Kurnyta, Maciej Henzel

**Affiliations:** 1 Faculty of Mechatronics and Aerospace, Military University of Technology, 00-908 Warsaw, Poland; maciej.henzel@wat.edu.pl; 2 Airworthiness Division, Air Force Institute of Technology, 01-494 Warsaw, Poland; artur.kurnyta@itwl.pl

**Keywords:** active magnetic suspension, jet engine, wireless measurement system, UAV

## Abstract

This paper presents laboratory results on the measurement system of a magnetic suspension bearing system for a jet engine rotor of an unmanned aerial vehicle (UAV). Magnetic suspension technology enables continuous diagnostics of a rotary machine and eliminates of the negative properties of classical bearings. This rotor-bearing system consists of two radial magnetic bearings and one axial (thrust) magnetic bearing. The concept of the bearing system with a magnetically suspended rotor for UAV is presented in this paper. Rotor geometric and inertial characteristics were assumed according to the parameters of a TS-21 jet engine. Preliminary studies of the measurement system of rotor engines were made on a laboratory stand with homopolar active magnetic bearings. The measurement system consisted of strain gauges, accelerometers, and contactless proximity sensors. During the research, strains were registered with the use of a wireless data acquisition (DAQ) system. Measurements were performed for different operational parameters of rotational rotor speed, control system parameters, and with the presence of disturbance signals from the control system. In this paper, obtained operational characteristics are presented and discussed.

## 1. Introduction

In recent years, magnetic suspension systems have been used in practice to solve certain problems of classical bearing systems that occur during the operation and maintenance of machinery and other technical objects. This technology was successfully implemented for the rail industry in Japan and China. In the track and vehicle, permanent magnets or electromagnets were implemented, generating magnetic levitation forces and providing non-contact movement of the vehicle on the track [1].

Magnetic bearing technology has been adopted in other branches of industry as well. It was developed and commercialized due to its advantages, such as lack of lubrication and no mechanical contact between operational elements [2,3]. Immense efforts have been undertaken to expand the magnetic technology applicability in high-speed machinery due to its invaluable advantages in terms of friction loss reduction, for example, in turbomachinery, compressors, and generators, [4,5,6,7,8,9]. In comparison with classical mechanical bearings, magnetic ones possess many advantages, [6,10] such as low amplitudes of mechanical vibration, high durability, lack of tribological wears, and ability of the extended operational term at high speed [11,12,13]. These features of magnetic technology give the immense potential to become a key element in smart and intelligent machines, such as unmanned aerial vehicle (UAV) jet engines [13].

Using active and passive magnetic bearing technologies in high-speed machinery can result in overcoming the physical limitations of classical bearings. This technology decreases stiffness coefficients and increases the damping coefficients of radial bearings, which reduce the value of critical rotor speed. Active magnetic bearings allow precise control of the rotor position and movement and enable “on-line” diagnosing, monitoring, and identification of high-speed machines. Thus, passive magnetic bearings can increase the damping coefficient of the bearing system for high rotor speed. In contrast to active bearings, passive ones do not consume electric energy. This technology uses magnets or superconductors to suspend the rotor of high-speed machinery. The concept of the structurally simple and operationally robust suspension of high-speed rotors in electrodynamic passive magnetic bearings (EDPMB) was presented in [14].

Developing a useful diagnostic concept of the rotor-bearing system is very important in the maintenance process of high-speed rotary machinery, including UAV jet engines. The accurate selection of the high-speed rotor-bearing system is continued with the design process. It is usually based on analysis of the load capacity, durability, bearing operating conditions, and experimental fatigue models of cooperating pairs (used to determine the bearing durability). However, these systems (without former change symptoms) are often damaged [15]. Therefore, an important issue is the development of modern and advanced methods of high diagnosis susceptibility for bearing system analyses. Rotating machinery monitoring methods are well established as well as detection algorithms for failure detection. Generally, rotary machinery monitoring systems are usually based on vibration signal analysis. These signals are essential and vital fault sources and contain enough information about operating states of rotating machinery [16]. The application of magnetic suspension technology in the bearing system of high-speed machine rotors enables one to measure vibrations with the use of eddy current sensors, which are a necessary control system element. Therefore, the possibility of using magnetic bearings creates an additional monitoring and diagnosis ability in comparison to classical rotary machines.

The structure of the diagnosis system correlates closely with the analyzed system, and only its elements have a universal character. Therefore, it is necessary to carry out measurements to establish a database (background) of typical operational parameters of the undamaged machine. Deviation from the initial parameters in the monitoring process constitutes the primary database for evaluating the technical conditions of rotating machinery. Dedicated condition indicators (CIs) are usually developed based on vibration signals, rotational speed values, local temperature gradients, and strain measurements. Changes in those CIs indicate damage progression. In magnetic bearings, the control signals (e.g., control current, air gap value) can also be used to monitor their operational conditions only after proper post-processing.

Preliminary studies of the monitoring system of the UAV jet engine rotor are presented in this paper. In the first section, the production of the magnetically suspended rotor of a small jet engine of a UAV is introduced. It consists of active and passive magnetic bearings as well. The second section presents a measurement system dedicated to the rotor-bearing system. Subsequently, a laboratory stand is presented. Finally, some conclusions and remarks are provided.

## 2. Design of the Magnetically Suspended Rotor of a UAV

Magnetic suspension technology allows the effective reduction of vibration amplitude and the elimination of friction force and negative operational properties of the classical bearings. It is estimated that the efficiency of magnetic suspension systems is 10% higher than for classical bearing systems, for example, by eliminating critical speeds [17]. Moreover, this solution increases the durability and the reliability of the bearing system and increases the operating range of the shaft’s rotational speeds.

Magnetic bearings are classified into two categories, namely passive and active bearings. Passive magnetic suspensions use permanent magnets or superconductors. They do not require a closed-loop control system. On the other hand, active magnetic suspensions use electromagnets to generate magnetic forces and require a feedback control signal (the open-loop control system is unstable) [18].

The fundamental element of an active magnetic bearing is an electromechanical actuator that generates a controlled force between cooperation pair–rotor and stator. There are four types of electromechanical actuators: heteropolar and homopolar electromechanical actuators, homopolar electromechanical actuators with permanent magnets, and homopolar electromechanical actuators with electromagnets [19].

**Figure 1:**
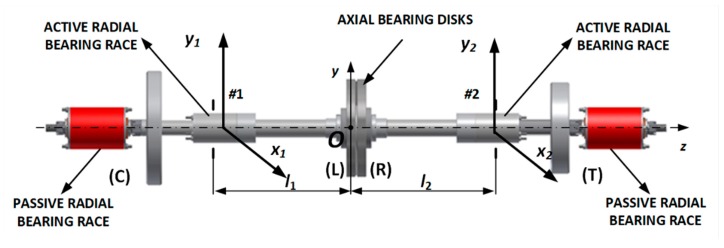
Magnetic suspension system of an unmanned aerial vehicle (UAV) rotor.

Figure 1 shows the design of the magnetic suspension system of a UAV rotor with active and passive magnetic bearings. Passive solutions effectively damp vibration amplitudes for high rotor speeds, without consuming electric energy. Radial active magnetic bearings generate radial forces in two perpendicular radial axes. The first active magnetic bearing #1 is controlled in the *x*_1_ and *y*_1_ axes’ coordinates. The second active radial magnetic bearing #2 is controlled in the *x*_2_ and *y*_2_ axes’ coordinates. The axial position in the *z*-axis (i.e., along the shaft axis) is controlled by axial forces generated by a thrust magnetic bearing. In total, there are five axes, *x*_1_, *y*_1_, *x*_2_, *y*_2_, and *z*, that are controlled by active magnetic bearing systems [10,18,20]. Radial active magnetic bearings suspend the rotor for low rotor speed, whereas passive ones suspend the rotor in the high-speed range. In this way, less energy is consumed than in the case of using only an active solution.

The designed magnetic suspension system for the UAV engine was developed for the rotor parameters corresponding to the TS-21 engine. This construction works as a starter on Mig-23 and Mig-27 aircraft, as well as Su-7, Su-20, and Su-22 aircraft. The TS-21 engine is driven by an electric starter with about 3 kW of power. The engine characterizes 1 kN thrust force, 60 ÷ 80 kW power, and 50,500 rpm. The rotor shaft has a 1.22 kg mass and is 15 mm in diameter.

In Figure 1, the elements marked as (C) and (T) are mass equivalents of the TS-21 compressor and turbine. The symbol *O* denotes the center of mass, which is located 178 mm (*l*_1_) from the “left” active radial bearing #1 and 191 mm (*l*_2_) from the “right” active radial bearing #2.

**Figure 2:**
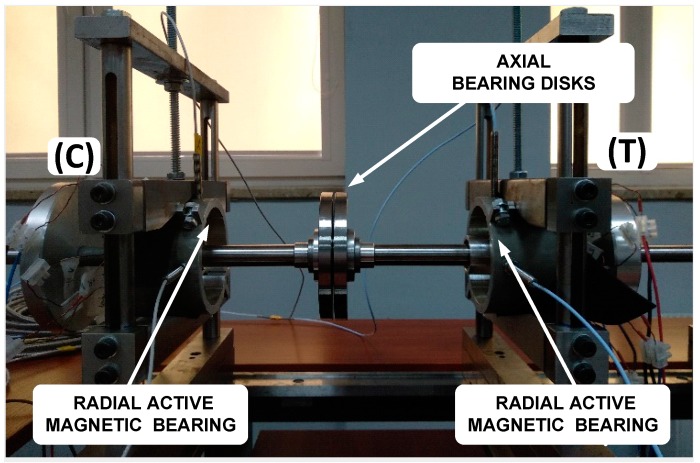
Laboratory stand with the magnetic suspension system of a UAV rotor.

Figure 2 presents the laboratory stand with the magnetic suspension system. The radial active magnetic bearings are set in the universal grips, and the axial magnetic bearing is dismounted. Air gaps in the radial and axial magnetic bearings are equal to 0.4 mm and 0.5 mm, respectively. Thus, the operating point current for each bearing is 5, and the maximum current for each bearing is 10 A, whereas axial one has 80 coils. Viscoelastic parameters of active magnetic bearings depend on control law parameters as well as current stiffness coefficient and displacement stiffness coefficient, being 3.74 × 10^5^ N/m and 1168 Ns/m, respectively. The axial bearing was designed to carry axial loads of 1.5 kN, because of the engine thrust force, whereas radial bearing was designed to maintain a 100 N load. Mass equivalents of compressor and turbine are marked as (C) and (T), as shown in Figure 1. Eddy current sensors are located in the electromagnet covers. Signals from these sensors provide information about rotor position for the closed-loop control system and could be useful for the diagnosis system as well.

**Figure 3:**
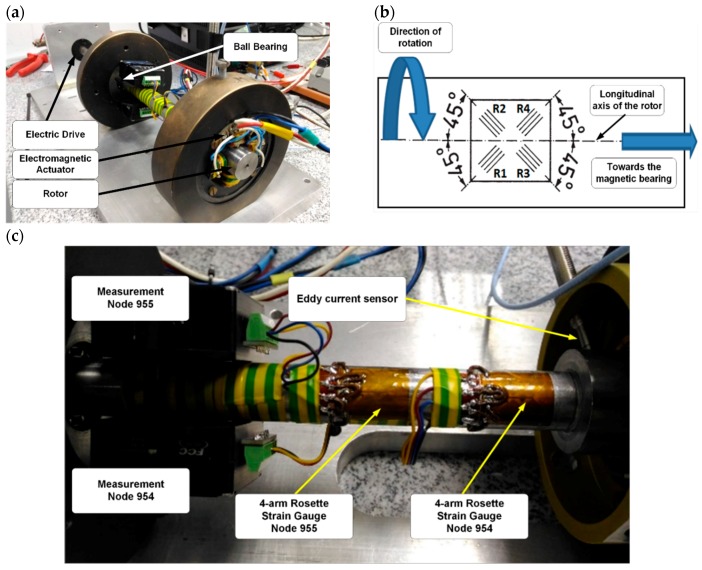
Laboratory stand of the active magnetic bearing with sensors: (**a**) laboratory model, (**b**) strain gauge configuration, (**c**) strain gauges with measurement nodes.

Due to the high level of complexity of the laboratory stand shown in Figure 2, preliminary qualitative studies were carried out on the system with only one active magnetic bearing and one ball bearing, presented in Figure 3. It consisted of an electromechanical actuator, rotor, control and data acquisition system, and an electric drive. The rotor was supported on one side by a classical ball bearing and on the other, by the magnetic bearing. The drive torque was transmitted to the rotor suspended by the magnetic bearing. For that bearing system, a measurement system was designed as a proof-of-concept. It consisted of eddy current sensors, which were a part of the control system and strain sensors.

In Figure 3b, the four-arm rosette strain gauge is illustrated with the orientation of its measuring grids (R1 ÷ R4) in comparison to the longitudinal axis of the rotor. The location of individual measurement system elements is shown in Figure 3c. In the next part of the paper, a laboratory stand with measurement system is described. Rotor deformation and rotor displacement are measured by strain gauges and eddy current sensors, respectively.

## 3. Measurement System

Bearing monitoring problems in turbomachinery, in the jet engines, currently represent significant issues. Monitoring systems should enable to detect defects in the early stages, before the breakdown of the whole system or the occurrence of further damage. Unfortunately, due to bearing operational conditions, monitoring of these parameters is not a simple task. Bearings are often located in places with difficult access. Another critical issue is the construction of moving engine parts, which make the use of even a simple cable connection impossible. It becomes necessary to use a wireless connection between sensors and recording modules. However, the use of wireless sensors can be made difficult due to the electromagnetic noise in a broad frequency spectrum that may occur during engine operation [13].

The laboratory stand provided a wireless data acquisition system to measure rotating shaft strain. Sensors for monitoring the strain under forced rotation were bonded on the shaft in two cross-sections, as shown in Figure 3. Measurement nodes with a transmitter for wireless telemetry were mounted near the ball bearing. Strain measurements were performed in the system build-up of the Wheatstone bridge, amplifier, and signal transmission circuit.

**Figure 4:**
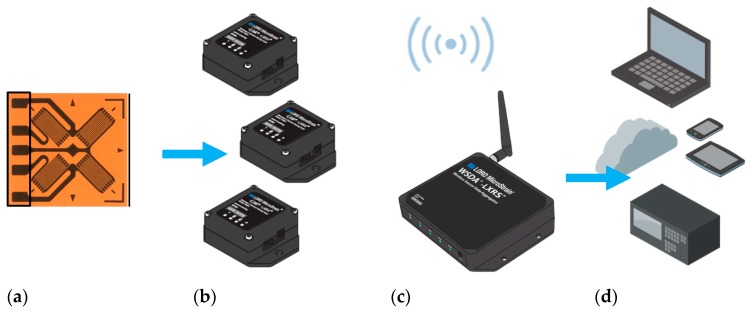
Diagram of the measurement system [13]: (**a**) strain gauge in a full-bridge configuration as a sensor, (**b**) wireless nodes, (**c**) gateway, (**d**) software.

The measurements of the shaft strains were performed for two cross-section areas (Nodes 954 and 955). Strain gauges operated first as a full-bridge and then as a quarter-bridge configuration, connected to the measurement system, as shown in Figure 4. The strain signal was measured and conditioned by nodes and wirelessly transferred to the gateway access point connected to the PC computer with dedicated software. A small, low-power analog sensor node SG-Link^®^ and the gateway WSDA^®^-200-USB from LORD MicroStrain^®^ Sensing Systems (Williston, VT, USA) were used for wireless measurements. Measurements were performed in two independent channels, namely Nodes 954 and 955. The grid rosettes for measuring the compress and stretch forces of the shaft in the longitudinal direction were in the range of ±45 degrees. Then, the strain gauge R1 was used to measure values of the local strains [21]. In this bridge configuration, the other Wheatstone’s bridge arms were supplemented by precision resistors with a resistance of 350 Ω ± 0.1%. The data acquisition system worked with 32 µs accuracy synchronization in a wide wireless range. Conducted measurements allowed to characterize the magnetically suspended rotor by comparing the local strain level in the middle and front parts of the shaft at various rotational speeds. Additionally, the natural feature of that type of bearing system is lack of torsion forces on the rotor. In that case, vibration components can be obtained directly on the rotor by using the above full-bridge strain gauge configuration.

**Figure 5:**
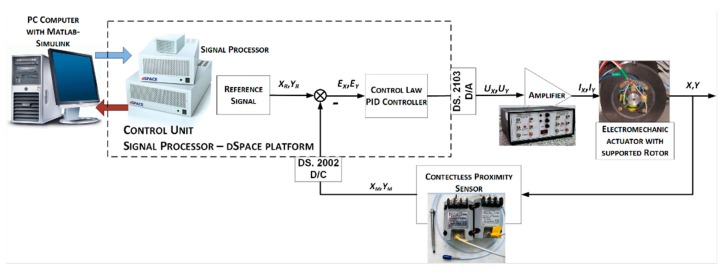
Active magnetic bearing control system.

The laboratory stand of the magnetic suspension system was also equipped with contactless position sensors to measure the rotor displacement in the air gap. These sensors were a part of the control system, as shown in Figure 5. The laboratory stand consisted of a control unit, proportional–integral–derivative PID controller, amplifier, electromechanical actuator with the supported rotor, and contactless eddy current sensors.

**Figure 6:**
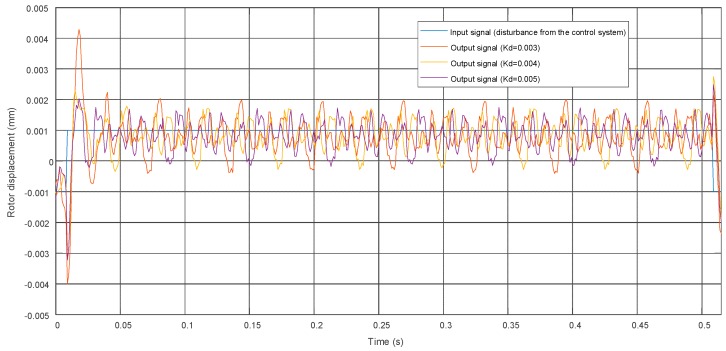
Time characteristics of the control systems with proportional-derivative PD controller for different *K_d_* values and constant *K_p_* equal to 1.75.

The control unit was based on a PC and the dSpace platform (dSpace GmbH, PaderbornyGermany) with input/output modules with 16-b A/D and D/A converters. The PID controller was designed in MATLAB Simulink 2010a software (Mathworks, Natick, MA, USA) and then implemented in the dSPACE platform. The control signals from the controller were transferred to two-channel bipolar amplifiers. The amplifiers supplied magnetic bearing electromagnets. Control current signals caused magnetic flux variations, which changed the rotor position. These displacements were measured by contactless eddy current sensors from the Bently Nevada company (Minden, NV, USA). Measured signals were transferred to the control unit via the input module. In this way, the feedback loop was obtained. Contactless proximity sensors can measure the rotor position in the range of 2 mm with an accuracy of 1 µm. Sample characteristics of the control system are presented in Figure 6.

The time characteristics of the control systems with the proportional-derivative PD controller are presented in Figure 6. The proportional parameter of the PID controller was marked as *K_p_* and was constant during the presented measurement, whereas the derivative parameter of the PID controller was marked as *K_d_*. The reference signal had the form of a rectangular signal acting on the *Ox* axis with amplitude and frequency equal to 0.01 V and 1 Hz, respectively. Later in the paper, this reference signal is called disturbance from the control system acting on the *Ox* or *Oy* axes. Other signals depicted in the figure present outputs from the control systems with the PD controller in the form of rotor displacements. In this figure, the vibration amplitude of the rotor can be observed. As the derivative parameter of controller *K_d_* increases, the vibration amplitude decreases. The system behaves like an oscillator with one degree of freedom and its transfer function reads as follows:

(1) $$ms^{2}+k_{i}K_{d}s+K_{p}k_{i}-k_{x}=0,$$

where *m* denotes rotor weight, current stiffness coefficient is described by *k_i_*, and displacement stiffness coefficient is expressed by *k_x_*. This equation has the following solutions *p*_1,2_:

(2) $$p_{1,2=-\omega_{0}\zeta \pm i\omega_{0}\sqrt{1-\zeta^{2}}},$$

where $\omega_{0}$ denotes the eigen frequency of the system, $\omega_{0}$ = 3.2/(*t_r_ζ*), *t_r_* is the settling time, and *ζ* is the dimensionless damping factor.

The location of the closed-loop system poles depends on settling time *t_r_* and the dimensionless damping factor *ζ*. When the poles *p*_1_ and *p*_2_ are known, PD controller parameter values can be calculated from the following relations:

(3) $$K_{d}=\frac{\left(-p_{1}-p_{2}\right)m}{k_{i}},$$

(4) $$K_{p}=\frac{p_{1}p_{2}m+k_{x}}{k_{i}}.$$

The above characteristic Equation (1) can be expressed as a differential equation of oscillatory motion:

(5) $$m\frac{d^{2}y}{dt^{2}}+c\frac{dy}{dt}+ky=0.$$

Comparing values of the equation parameters, the stiffness coefficient is described by the following:

(6) $$k=K_{p}k_{i}-k_{x}.$$

Finally, the damping coefficient reads as follows:

(7) $$c=k_{i}K_{d}$$

From the above considerations about using a PD controller, it is possible to have an effect on stiffness and damping coefficients of the entire active magnetic bearing system.

**Figure 7:**
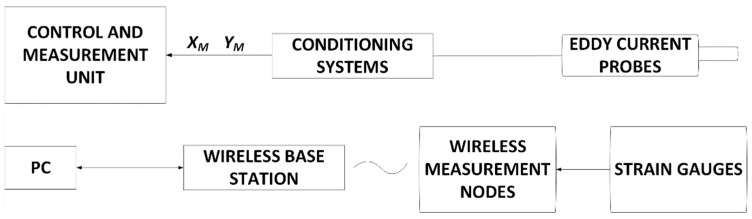
Flowchart with the overall measurement process.

In conclusion, a measurement system for active magnetic bearings consists of strain gauges and eddy current sensors to measure strains and rotor displacements, respectively. The flowchart of the overall measurement process is presented in Figure 7. At the laboratory stage of the study, comparison results were obtained from strain gauges placed on the rotor surface. The analysis of research results allows the development of the monitoring and diagnosis system for a bearing system for a UAV engine rotor, equipped with passive and active magnetic bearings.

## 4. Results

The rotary system with an active magnetic bearing, as shown in Figure 3, was investigated to obtain its dynamic behavior with the developed measuring system. This system enabled the continuous measurement of the magnetic bearing operational parameters, such as rotating shaft strains, rotor displacements, and vibrations. During studies, strain measurements with full bridge and quarter bridge were registered by a wireless data acquisition (DAQ) system. The sensors were located in two cross-sections of the magnetically suspended shaft, that is, one in the middle (Node 955) and the second near the magnetic bearing (Node 954). Two different bridge configurations for strain gauge were utilized to measure the local uniaxial strain with a quarter bridge and quasi-vibrations from the rotational motion with a full bridge. In a classical bearing system, a full-bridge configuration allows the measurement of torsion strain, but in the magnetic bearing system, this component is absent.

Strain measurements were conducted for both cross-sections simultaneously for constant parameters of the control system and disturbance signals acting on the *Ox* and *Oy* axes. These disturbances were generated by the control system. Strain time-domain signals were acquired using a 512 Hz sampling frequency, for approximately 20 seconds of stable rotational speed. For each rotational speed, three measuring series were made with no disturbance, disturbances added in the *Ox* axis, and disturbances added in the *Oy* axis. Fast Fourier transform (FFT) analysis was performed for time-domain signals and the frequency characteristics are presented below.

**Figure 8:**
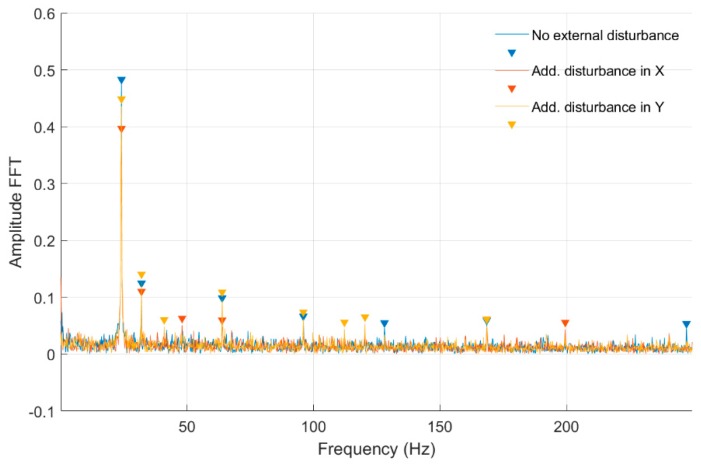
Fast Fourier transform (FFT) of strain-level measurement for constant motor speed equal to 25 rev/s with disturbance from the control system for a quarter-bridge configuration (Node 954).

**Figure 9:**
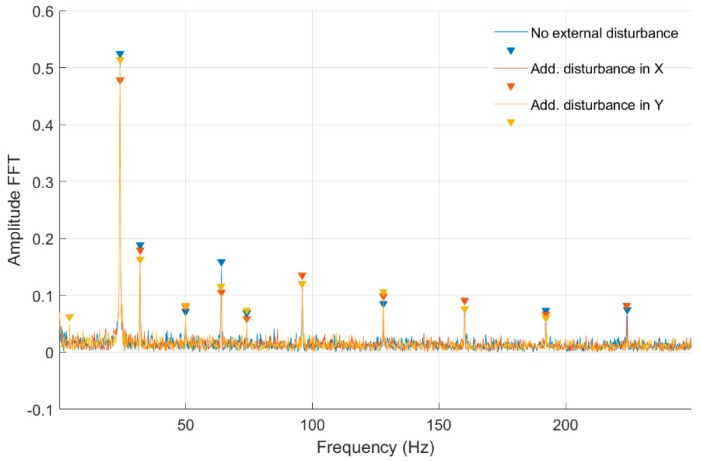
FFT of strain-level measurement for constant motor speed equal to 25 rev/s with disturbance from the control system for a quarter-bridge configuration (Node 955).

In Figure 8 and Figure 9, fast Fourier transform (FFT) characteristics of the rotating shaft strain measured for a constant motor rotary speed equal to 25 rev/s for a quarter-bridge configuration at both shaft cross-sections, Nodes 954 and 955, are presented, respectively. These characteristics were registered without and with disturbance from the control system. Measurement with no disturbance is marked by a blue line, and measurements with disturbance added to the control signal in the *Ox* and *Oy* axes are indicated by red and orange lines, respectively. In Table 1, the list of significant values from the characteristics shown in Figure 8 and Figure 9 is presented. Cells with peaks from the sampling frequency are marked in blue.

**Figure 10:**
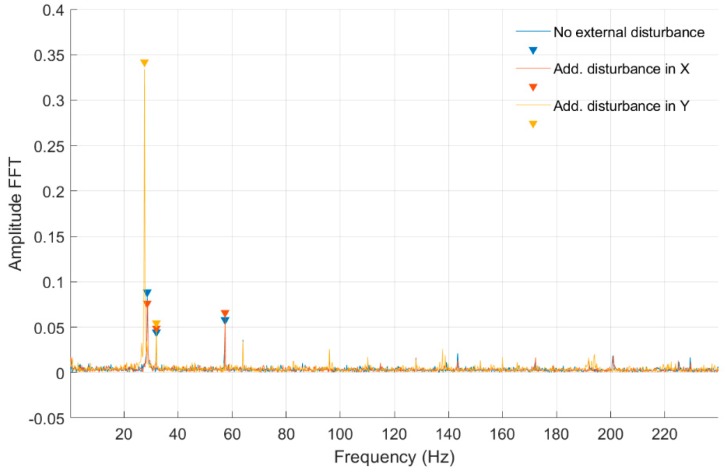
FFT of strain-level measurement for constant motor speed equal to 30 rev/s with disturbance from the control system for a full-bridge configuration (Node 954).

**Figure 11:**
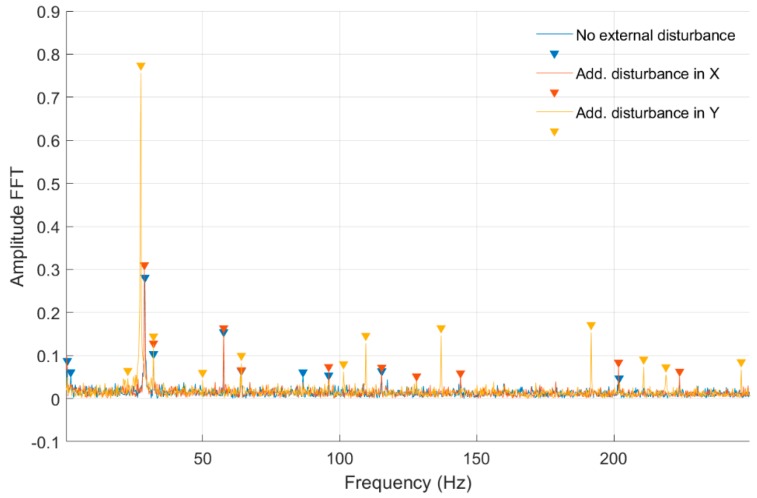
FFT of strain-level measurement for constant motor speed equal to 30 rev/s with disturbance from the control system for a quarter-bridge configuration (Node 954).

In Figure 10 and Figure 11, fast Fourier transform (FFT) characteristics of the rotating shaft strain measured at the shaft front cross-section (Node 954) for a constant motor rotary speed equal to 30 rev/s for both full- and quarter-bridge configurations are presented, respectively. These characteristics were registered without and with disturbance from the control system. Significant values from obtained characteristics are presented in Table 2.

**Figure 12:**
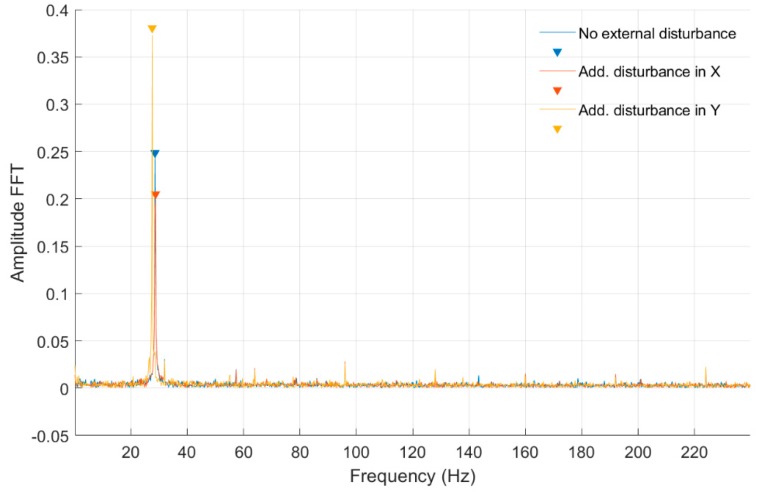
FFT of strain level measurement for constant motor speed equal to 30 rev/s with disturbance from the control system for a full-bridge configuration (Node 955).

**Figure 13:**
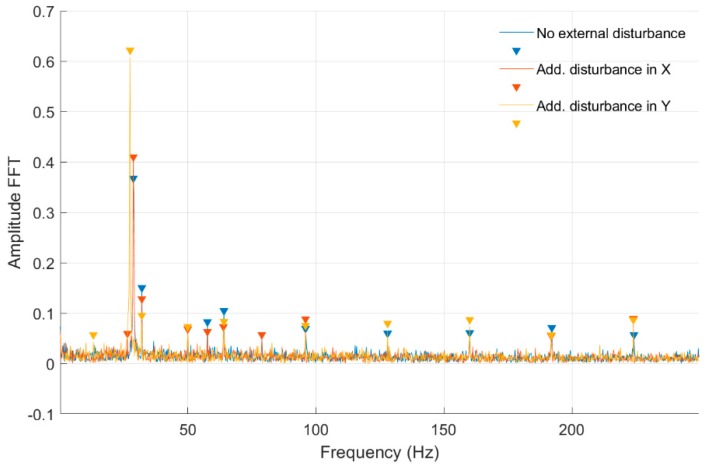
FFT of strain-level measurement for constant motor speed equal to 30 rev/s with disturbance from the control system for a quarter-bridge configuration (Node 955).

In Figure 12 and Figure 13 and Table 3, the same set of characteristics as above are presented, but from the middle shaft cross-section.

**Figure 14:**
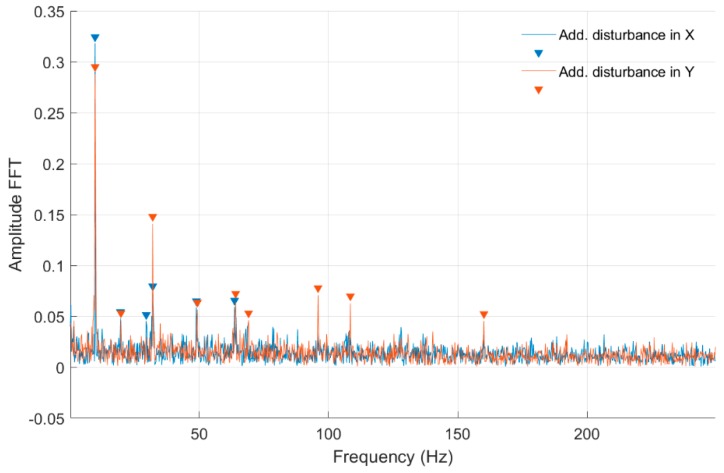
FFT of strain-level measurement for constant motor speed equal to 7 rev/s with disturbance from the control system for a quarter-bridge configuration (Node 954).

**Figure 15:**
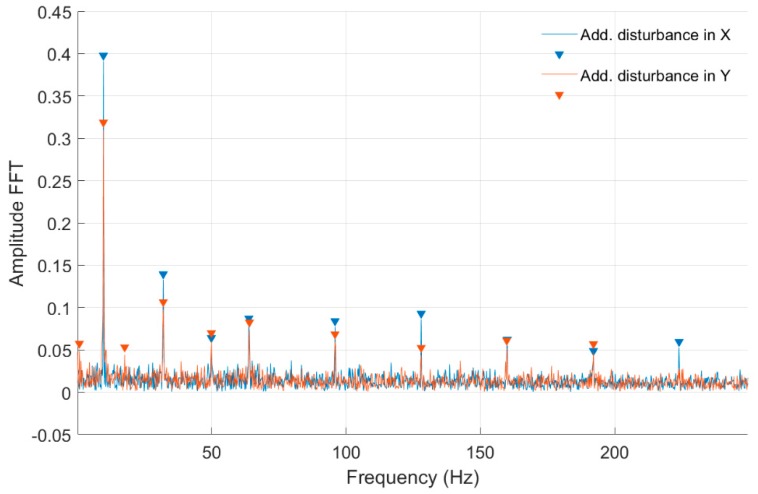
FFT of strain-level measurement for constant motor speed equal to 7 rev/s with disturbance from the control system for a quarter-bridge configuration (Node 955).

**Figure 16:**
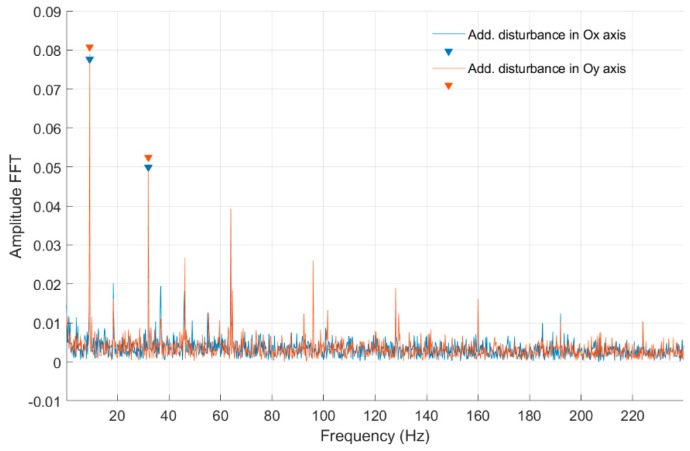
FFT of strain-level measurement for constant motor speed equal to 7 rev/s with disturbance from the control system for a full-bridge configuration (Node 954).

**Figure 17:**
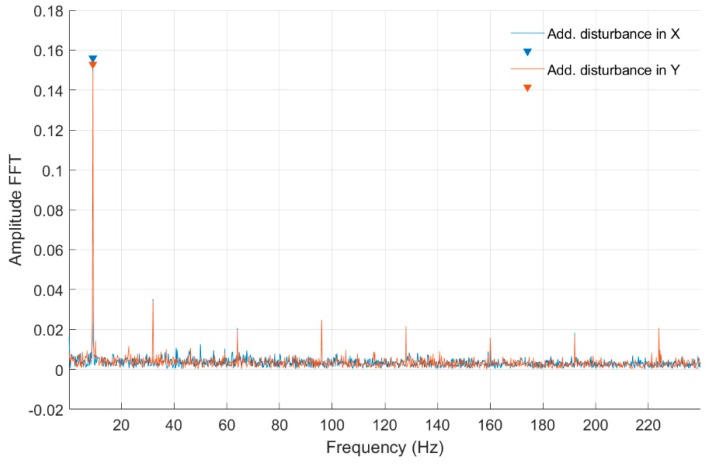
FFT of strain-level measurement for constant motor speed equal to 7 rev/s with disturbance from the control system for a full-bridge configuration (Node 955).

In Figure 14, Figure 15, Figure 16 and Figure 17 and Table 4, fast Fourier transform (FFT) characteristics of the rotating shaft strain measured for a constant motor rotary speed equal to 7 rev/s for quarter- and full-bridge configurations at both front and middle shaft monitoring points are presented.

## 5. Discussion

Presented test results were carried out for various rotational speeds of the magnetically suspended rotor and constant values of controller parameters. Proportional and differential gains of the controller were set to 1.75 and 0.003, respectively, and the amplitude of disturbance was set to 0.01 V with a frequency of 1 Hz. Differences in results for the quarter- and full-bridge strain measurements are attributed to various types of strain state to which the bridge configuration is sensitive, as one or four grids are actively used to obtain data.

In Table 1 were presented the FFT amplitudes and corresponding frequencies of strain-level measurements for an engine speed equal to 25 rev/s without and with external disturbance measured for Nodes 954 and 955 for a quarter-bridge configuration. Cells with the frequency of 24.14 Hz correspond to engine speed, whereas cells marked by blue peaks are the artefacts from the sampling frequency. For the measurement with no disturbance for the front shaft cross-section, the frequency response is clear with no additional peaks. For measurements with external disturbance in the *Ox* and *Oy* axes for Node 954, peaks at the frequencies of 48 Hz and 41 Hz occurred, respectively. In the case of Node 955 measurements without and with external disturbance, peaks at 49.95 and 74.09 Hz occurred. It can be clearly seen that an added disturbance signal gives an additional frequency response for both cross-sections. Both peak amplitudes are higher at the same frequencies for Node 955 (middle cross-section of the shaft) in comparison with Node 954 (front cross-section) in both axes. However, the ratio of FFT amplitudes for measurements without and with disturbance is lower for the front cross-section and is 0.81 for *Ox* and 0.92 for *Oy* axes, respectively. For the middle cross-section, 0.9 for the *Ox* axis and 0.97 ratios were obtained. That suggests the higher impact of an added disturbance in both axes for the cross-section closer to the magnetic bearing.

In Table 2 and Table 3 were presented the FFT amplitudes and corresponding frequencies of strain-level measurements for an engine speed equal to 30 rev/s without and with external disturbance for Node 954 for quarter- and full-bridge configurations. Cells with the frequency of approximately 28 Hz correspond to engine speed. For the measurement with full-bridge configuration, the frequency characteristics are much clearer with a smaller number of peaks than in the case of the quarter-bridge configuration. The FFT amplitude ratio for measurements without and with disturbance in the *Ox* axis is similar for the first harmonic in both shaft cross-sections and is equal to 1.10. However, in the second harmonic, the ratio is 0.72 and 1.05 for the middle and front measuring points. Regarding the added disturbance in the *Oy* axis, the amplitude ratio differs significantly with values of 1.68 for the middle and 2.74 for the front shaft cross-sections. The analysis for full-bridge measurements reveals a similar effect with added disturbance in the *Ox* and the *Oy* axes. Here, the influence of added disturbance in the *Oy* axis caused the further increase of the FFT amplitude ratio to values of 1.53 for the middle and 3.84 for the front measuring points. It can also be seen that the first harmonic frequency with added disturbance in the *Oy* axis is slightly decreased in comparison to those with no disturbances and added in the *Ox* axis. The same phenomena can be observed for quarter-bridge measurements, although those were performed separately. That decrease in the frequency characteristic was absent for lower rotational speeds. The specific source of that effect requires further investigation. It is probable that additional deflection modes occurred for the shaft at that speed, when a disturbance in the *Oy* axis was present. An higher FFT amplitude with further frequency harmonics was measured for the quarter-bridge configuration compared with the full-bridge configuration for that rotational speed. Again, the shaft cross-section closer to the magnetic bearing was influenced more than its middle part in the presence of disturbances.

In Table 4 were presented the FFT amplitudes and corresponding frequencies of strain-level measurements for a low engine speed equal to 7 rev/s with external disturbance for Nodes 955 and 954 for quarter- and full-bridge configurations. For that rotational speed, no noticeable effects were detected for disturbances in the *Ox* and *Oy* axes in both bridge configurations. Several frequency peaks were seen for the strain gauge connected to Node 954 in the front of the shaft, which suggests a higher level of strain and vibrations on frequencies above and even below the rotational speed. Additionally, higher harmonics were visible for the full-bridge configuration at that point. In classical bearing diagnostics, that is an indicator of degradation. With the magnetic bearing, it can be a sign of a rotational speed that is too low to ensure a suitable level of self-centering of the shaft to the working point.

In summary, the performed measurements revealed that the strain gauge monitoring point should be located near the magnetic bearing, as the presence of induced disturbances influenced more that part of the rotating shaft. Because of the lack of physical contact between rotor and stator, the classical approach for bearing diagnostics with the use of accelerometers would not be as efficient. The full-bridge configuration gave a clearer indication of induced disturbances with a lower level of noise in the frequency spectrum. Disturbance in the *Oy* axis impacted the frequency characteristics more than the equal disturbance in the *Ox* axis. This is caused by the fact that the *Oy* axis stabilizes vertically and provides the lifting force for the rotating shaft. Therefore, even in a static condition, forces generated by the lower and upper coils are not equal, as in the *Ox* axis.

## 6. Conclusions

This paper presented preliminary studies of a measurement system dedicated to UAV engine rotors. Moreover, the new concept of a rotor suspension system with magnetic bearings for a mini turbojet engine rotor was described. In the proposed support system, both active and passive magnetic bearings were introduced. Magnetic suspension technology allows the efficient reduction of the transverse vibration amplitude and the negative performance features of a classical bearing system [7]. Additionally, this technology enables continuous diagnostics of rotor engines.

In this paper, a wireless measuring system for the rotary machine was introduced. It consisted of strain sensors and measuring modules with a transmitter for wireless telemetry. Measurements of strain gauge were performed in the system build-up of the Wheatstone bridge, amplifier, and signal transmission circuit. During studies, strain measurements with strain gauges located in two cross-sections of the magnetically suspended shaft were registered. Measurements were made for the different rotational speeds of the rotor, different control system parameters, and various disturbances acting on the *Ox* and *Oy* axes. Registered characteristics presented strain amplitude in two cross-sections and fast Fourier transform of the strain measurements for constant motor speed, without and with disturbance. The measurement revealed a higher amplitude level with an increase of rotational speed. Additionally, disturbances in the *Oy* axis were more noticeable compared with the data with no disturbance and those added in the *Ox* axis, especially in the front cross-section monitoring point.

Developed laboratory stands with the magnetically suspended rotor and the positive effect of preliminary studies have opened new perspectives for development work associated with the monitoring system of UAV rotor engines. Results obtained during studies are the basis for developing an identification and monitoring system of a rotor supported by magnetic bearings. The next stage of studies includes an attempt to compare measurements from strain gauges and eddy current sensors to indicate phenomena related to incorrect rotor operation using sensors integrated with the object.

## Figures and Tables

**Table 1:** List of parameters read from the characteristics shown in Figure 8 and Figure 9.

No external force for Node 954						
FFT Amplitude	0.474	0.116	0.090		0.058	
Frequency (Hz)	24.14	32.05	63.90		95.95	
Additional force in *Ox* axis for Node 954						
FFT Amplitude	0.385	0.098	0.051		0.048	
Frequency (Hz)	24.14	32.05	48.08		63.90	
Additional force in *Oy* axis for Node 954						
FFT Amplitude	0.437	0.128	0.048		0.097	
Frequency (Hz)	24.14	32.05	41.00		63.90	
No external force for Node 955						
FFT Amplitude	0.514	0.178	0.062	0.149	0.059	0.110
Frequency (Hz)	24.14	32.05	49.95	64.10	74.09	95.95
Additional force in *Ox* axis for Node 955						
FFT Amplitude	0.466	0.167	0.068	0.93	0.047	0.123
Frequency (Hz)	24.14	32.05	49.95	64.10	74.09	95.95
Additional force in *Oy* axis for Node 955						
FFT Amplitude	0.050	0.502	0.151	0.070	0.104	0.062
Frequency (Hz)	4.16	24.14	32.05	49.95	63.90	74.09

**Table 2:** List of parameters read from the characteristics shown in Figure 10 and Figure 11.

No external force for Node 954, full bridge				No external force for Node 954, quarter bridge								
FFT Amplitude	0.087	0.043	0.056	FFT Amplitude	0.082	0.056	0.276	0.099	0.149	0.060	0.056	0.049
Frequency (Hz)	28.60	32.00	57.40	Frequency (Hz)	0.62	1.87	28.93	32.05	57.65	64.10	86.58	95.95
Additional force in *Ox* axis for Node 954, full bridge				Additional force in *Ox* axis for Node 954, quarter bridge								
FFT Amplitude	0.074	0.047	0.064	FFT Amplitude	0.303	0.122	0.157	0.060	0.067	0.066		
Frequency (Hz)	28.60	32.00	57.40	Frequency (Hz)	28.72	32.05	57.65	64.10	95.95	115.30		
Additional force in *Oy* axis for Node 954, full bridge				Additional force in *Oy* axis for Node 954, quarter bridge								
FFT Amplitude	0.334	0.047		FFT Amplitude	0.047	0.756	0.127	0.043	0.082	0.062	0.128	
Frequency (Hz)	27.60	32.00		Frequency (Hz)	22.69	27.47	32.05	49.95	64.10	101.35	109.48	

**Table 3:** List of parameters read from the characteristics shown in Figure 12 and Figure 13.

No external force for Node 955, full-bridge		No external force for Node 955, quarter bridge								
FFT Amplitude	0.244	FFT Amplitude	0.361	0.143	0.076	0.098	0.050	0.063		
Frequency (Hz)	28.60	Frequency (Hz)	28.72	32.05	57.65	64.10	78.88	95.95		
Additional force in *Ox* axis for Node 955, full-bridge		Additional force in *Ox* axis for Node 955, quarter bridge								
FFT Amplitude	0.199	FFT Amplitude	0.052	0.402	0.120	0.062	0.055	0.065	0.049	0.080
Frequency (Hz)	28.80	Frequency (Hz)	26.43	28.72	32.05	49.95	57.65	63.90	78.88	95.95
Additional force in *Oy* axis for Node 955, full-bridge		Additional force in *Oy* axis for Node 955, quarter bridge								
FFT Amplitude	0.373	FFT Amplitude	0.043	0.608	0.081	0.059	0.069	0.061		
Frequency (Hz)	27.60	Frequency (Hz)	13.11	27.47	32.05	49.95	64.10	95.95		

**Table 4:** List of parameters read from the characteristics shown in Figure 14, Figure 15, Figure 16 and Figure 17.

Additional force in *Ox* axis for Node 954, full bridge				Additional force in *Ox* axis for Node 954, quarter bridge							
FFT Amplitude	0.076	0.047	0.020	FFT Amplitude	0.318	0.048	0.045	0.073	0.059	0.059	
Frequency (Hz)	9.20	32.00	18.40	Frequency (Hz)	9.78	19.56	29.56	32.05	48.91	63.69	
Additional force in *Oy* axis for Node 954, full bridge				Additional force in *Oy* axis for Node 954, quarter bridge							
FFT Amplitude	0.079	0.047	0.016	FFT Amplitude	0.288	0.046	0.141	0.057	0.065	0.046	0.071
Frequency (Hz)	9.20	32.00	18.40	Frequency (Hz)	9.78	19.77	32.05	49.33	64.10	69.10	95.95
Additional force in *Ox* axis for Node 955, full bridge				Additional force in *Ox* axis for Node 955, quarter bridge							
FFT Amplitude		0.153		FFT Amplitude	0.391	0.133	0.057	0.080	0.077		
Frequency (Hz)		9.20		Frequency (Hz)	9.78	32.05	49.95	63.90	95.95		
Additional force in *Oy* axis for Node 955, full bridge				Additional force in *Oy* axis for Node 955, quarter bridge							
FFT Amplitude		0.149		FFT Amplitude	0.049	0.310	0.044	0.098	0.061	0.074	0.060
Frequency (Hz)		9.20		Frequency (Hz)	0.83	9.78	17.69	32.05	49.95	64.10	95.95
